# Sequence Variations and Protein Expression Levels of the Two Immune Evasion Proteins Gpm1 and Pra1 Influence Virulence of Clinical *Candida albicans* Isolates

**DOI:** 10.1371/journal.pone.0113192

**Published:** 2015-02-18

**Authors:** Shanshan Luo, Uta-Christina Hipler, Christin Münzberg, Christine Skerka, Peter F. Zipfel

**Affiliations:** 1 Department of Infection Biology, Leibniz-Institute for Natural Product Research and Infection Biology, Hans-Knöll-Institute, Jena, Germany; 2 Friedrich-Schiller-University Hospital, Clinic of Dermatology and Allergology, Jena, Germany; 3 Friedrich-Schiller-University, Jena, Germany; University Medical Center Utrecht, NETHERLANDS

## Abstract

*Candida albicans*, the important human fungal pathogen uses multiple evasion strategies to control, modulate and inhibit host complement and innate immune attack. Clinical *C. albicans* strains vary in pathogenicity and in serum resistance, in this work we analyzed sequence polymorphisms and variations in the expression levels of two central fungal complement evasion proteins, Gpm1 (phosphoglycerate mutase 1) and Pra1 (pH-regulated antigen 1) in thirteen clinical *C. albicans* isolates. Four nucleotide (nt) exchanges, all representing synonymous exchanges, were identified within the 747-nt long *GPM1* gene. For the 900-nt long *PRA1* gene, sixteen nucleotide exchanges were identified, which represented synonymous, as well as non-synonymous exchanges. All thirteen clinical isolates had a homozygous exchange (A to G) at position 73 of the *PRA1* gene. Surface levels of Gpm1 varied by 8.2, and Pra1 levels by 3.3 fold in thirteen tested isolates and these differences influenced fungal immune fitness. The high Gpm1/Pra1 expressing candida strains bound the three human immune regulators more efficiently, than the low expression strains. The difference was 44% for Factor H binding, 51% for C4BP binding and 23% for plasminogen binding. This higher Gpm1/Pra1 expressing strains result in enhanced survival upon challenge with complement active, Factor H depleted human serum (difference 40%). In addition adhesion to and infection of human endothelial cells was increased (difference 60%), and C3b surface deposition was less effective (difference 27%). Thus, variable expression levels of central immune evasion protein influences immune fitness of the human fungal pathogen *C. albicans* and thus contribute to fungal virulence.

## Introduction

The human pathogenic yeast *Candida albicans* is frequently isolated from individuals with fungal infections. This dimorphic fungal pathogen causes superficial, as well as systemic, infections and is frequently isolated from patients who undergo immunosuppressive therapy or long-term catheterization [1,2,3]. In infected individuals, Candida is identified in blood, brain, eye, kidney, heart, as well as lung, liver, and spleen. The symptoms of Candida infection vary to a great extent and depend on the site of infection. Oral candidiasis develops characteristic thick, white lacy patches on top of a red base, which may induce inflammatory reactions on the tongue, as evidenced by the red color, sometimes even without any white coating [4]. Vaginal infections represent as white cheesy discharge, which irritates the internal and also the surrounding outer tissues [5]. Even with the use of anti-fungal therapy, both mortality and morbidity by *C*. *albicans* infections remain unacceptably high and over 75% of patients with systemic Candida infection die [6,7,8]. Moreover, the rate of treatment-resistant *C*. *albicans* strains has been steadily increasing, while vaccination development has remained challenging and lagging [9]. Therefore, a detailed characterization of *C*. *albicans* virulence factors is necessary not only for understanding the process of infection in detail but also for generating new and more effective anti-fungal compounds.

Complement is a central innate immune surveillance system, that acts as the first line of innate immune defense [10]. Upon infection the host complement system is activated within seconds [11] and the activated system generates several immune effector molecules which coordinate immune response, cell infiltration and have toxic effects on the target. Based on these toxic effects, activation and processing of this important defense system is tightly regulated by both fluid phase and surface associated regulators. The proper and coordinated action of these regulators is important for maintaining homeostasis and to efficiently protect the human organism from infections agents [12,13,14,15,16,17,18]. Thus, in order to survive and establish an infection the human fungal pathogen *C*. *albicans*, similar to other human pathogenic fungus, acquires human plasma proteins and complement regulators, including Factor H, FHL-1 (factor H like protein 1) and C4BP (C4 binding protein), as well as plasminogen [19,20,21,22]. Bound to the fungal surface, these human inhibitors maintain their regulatory activity, block complement effector function(s), opsonization and phagocytosis, inhibit cascade activation, as well as cascade progression, and further inhibits the damaging, toxic and inflammatory effector functions of the activated complement system [20,21,23]. At present, four Candida Factor H/FHL-1 binding proteins are identified, which are Gpm1, Pra1 [24,25], Hgt1p as well as Gpd2 [26,27]. Gpm1, Pra1 and Gpd2 also bind the coagulation protease and complement inhibitor plasminogen. In addition, seven other plasminogen binding proteins were identified on *C*. *albicans* surface via a proteome approach [28].

Many, even all infectious pathogens exploit host Factor H and plasminogen for immune evasion and for tissue penetration. In general terms, pathogenic microbes express host regulator binding proteins at their surface that bind several human immune inhibitors and preproteases, like Factor H or plasminogen [29,30,31,32,33]. Microbial immune evasion displays features of multiplicity and by redundancy. Apparently a single microbial immune evasion protein binds a unique repertoire of human proteins and immune regulators, e.g. Candida Pra1 binds Factor H, FHL-1, C4BP, C3, plasminogen, and blocks cleavage and activation of C3 by a preformed C3 convertase [25,34,35]. Furthermore, Candida Pra1 also binds to the human integrin receptors CR3 and CR4 which are expressed on leukocytes [36]. In addition, a pathogenic microbe expresses several host immune regulator binding proteins at the same time. Although the different microbial immune evasion proteins which attach the same host regulator, e.g. via the same or overlapping regions, lack common sequences motives [29,30,37]. The microbial Factor H binding proteins represent either highly polymorphic proteins, that display a high degree of sequence diversity, or they present as conserved genes, with no or even moderate sequence variation. Examples for the diverse group of microbial Factor H-plasminogen binding proteins, which display a high degree of sequence diversity, include the M protein encoding gene family of *S pyogenes*, and the PspC protein family of *S*. *pneumonia* [38,39,40,41]. Examples for the second group, with more conserved genes, are *CRASPs (complement regulator-acquiring surface proteins)* of *Borrelia* species and also *FHBP* of group B *N*. *meningitidis* isolates [42].

Currently, it is unclear whether the two Candida Factor H/plasminogen binding proteins Gpm1 and Pra1 represent conserved or polymorphic fungal immune evasion proteins and/or whether the expression levels of the two proteins vary among clinical Candida isolates. Therefore, we analyzed sequence variability and expression levels of the *GPM1* and *PRA1* genes in thirteen clinical *C*. *albicans* isolates. Here we show that the Candida *GPM1-* and *PRA1* are conserved genes and that their expression levels varied by 8.2 fold for Gpm1 (MFI 512 to 4192) or 3.3 fold for Pra1 (MFI 12393 to 41282) respectively. This difference in Gpm1-, Pra1 surface levels influenced fungal immune fitness in terms of human regulator binding, survival in complement active, Factor H depleted human serum, adhesion to human endothelial cells, as well as C3b/iC3b surface deposition. Thus surface levels likely in combination with sequence variations influence and adjust immune fitness of Candida/fungal isolates.

## Materials and Methods

### Study approval and collection of clinical *C*. *albicans* isolates

The study was approved by the Ethical Board of the Medical Department of the Friedrich Schiller University in Jena, Germany. Patients provided written consent for participation in the study. In total, thirteen strains were tested. The strains had been isolated from dermatological patients (Department of Dermatology, Jena, Germany), using FungiQuick swabs (Hain lifescience, Nehren, Germany). The swabs were incubated in Sabouraud glucose bouillon included in the FungiQuick test kit (t = 24 h, room temperature). Afterwards, the yeast containing bouillon was plated onto Sabouraud glucose agar plates and incubated for 24 h at 30°C. The obtained cultures were used for species identification by the API ID 32C test system (bioMerieux) according to instructions of the manufacturer (test results were read after 24 h and 48 h).

### *C*. *albicans* strains and growth conditions

The *C*. *albicans* wild type strains SC5314 [43] and different clinical isolates, which were isolated from different *C*. *albicans* infected patients were cultivated in YPD medium (2% (w/v) glucose, 2% (w/v) peptone, 1% (w/v) yeast extract) at 30°C. Yeast cells were collected by centrifugation and counted with a hemocytometer (Fein-Optik, Bad Blankenburg).

### Antibodies and anti-sera

Polyclonal Gpm1- or Pra1 anti-sera was raised in rabbits by immunization with purified recombinant Gpm1 or Pra1 protein [24,25]. Polyclonal rabbit Saps1/2/3 (Secreted aspartyl proteases) anti-serum was kindly provided by Prof. Bernhard Hube (Germany). Polyclonal goat anti Factor H was purchased from CompTech, polyclonal goat anti plasminogen from Acris, and polyclonal rabbit anti-C4BP was kindly provided by Prof. Anna M. Blom (Sweden). Alexa Fluor-647 labeled goat anti-rabbit and Alexa Fluor-488 labeled rabbit anti-goat, goat anti-rabbit were all obtained from Molecular Probes used as the secondary anti-sera for flow cytometry. Serum were collected from five healthy donors, pooled together, and stored at -80°C until use. Factor H depleted serum was prepared as described [44].

### Sequence analysis

The DNA sequences of *GPM1* and *PRA1* from thirteen different clinical isolates and the reference strain SC5314 were amplified by PCR, sequenced and compared. Briefly, different *C*. *albicans* yeast pellets were boiled at 99°C for 10 min in H_2_O with 300 rpm shaking, then centrifuged at 13000 rpm for 10 min to get the supernatant which contains chromosomal DNA. These chromosomal DNA were used as the template. The *GPM1* gene was amplified with forward primer: CCTTGCTCCAATTATCCTTTGAT, and reverse primer: CTACAAATCAAACCACACATCTA. Due to the long sequence, the *PRA1* gene was amplified using two pairs of primers (Forward 1: TGGAAAAGACCTTTGTTTGG, Reverse 1: TTAGATTTCGAGACGGTATA, and with Forward 2: CCAACCATAGTGATCAAACT, Reverse 2: TCTATAAAAAGATATCCATG) in order to get a precise sequence. The PCR products of both genes were first separated by the agarose gel electrophoresis, then the bands were visualized under the UV detector (GENE GENIUS 1, SYNGENE). These PCR products were purified by GFX purification kit (GE healthcare). The purified PCR products were used for sequencing with 3130x1 Genetic Analyzer using the above primers (AB applied Biosystems).

### Flow cytometry

To compare Gpm1 and Pra1 surface expression level on different *C*. *albicans* strains, each of the thirteen clinical isolates and wild type SC5314 were cultivated in YPD medium overnight at 30°C. Cells were washed and incubated with polyclonal Gpm1 or Pra1 anti-serum (1:200) for 30 min on ice, followed by an Alexa Fluor-647 labeled goat anti-rabbit as a secondary anti-serum for another 30 min on ice. After washing, samples were sonicated for 10min before the fluorescence signal was determined by flow cytometry (LSR II, BD bioscience).

For Factor H, plasminogen and C4BP binding the clinical *C*. *albicans* isolates and SC5314 strain were incubated with EDTA-NHS (1:3 diluted of NHS (normal human serum) in DPBS (Dulbecco’s, buffered saline), supplemented with 10 mM EDTA to inhibit the complement activation) for 30 min at 37°C, then *C*. *albicans* cells were pellet, and re-suspended in 1% BSA in DPBS for washing, then incubated with polyclonal goat anti Factor H, rabbit anti C4BP or goat anti plasminogen sera (1:200 diluted in 1% BSA in DPBS) for 30 min on ice, followed by Alexa Fluor-488 labeled rabbit anti-goat or goat anti-rabbit as secondary anti-serum for 30 min on ice. Then the samples were washed, sonicated and examined by flow cytometry.

### *C*. *albicans* survival assay

Different *C*. *albicans* clinical isolates as well as the reference strain SC5314 were cultivated in YPD medium overnight at 30°C. The yeast cells (1x10^4^/ml) were incubated with 10% Factor H depleted serum in Mg-EGTA buffer (20 mM/l HEPES, 144 mM/l NaCl, 7 mM/l MgCl_2_, 10 mM/l EGTA [pH 7.4]) (which only allows activation of the alternative pathway) for 60 min at 37°C. At the time point 0 and 60 min, 10 μl of each sample were collected, transferred onto a YPD agar plate, cultivated for 2 days at 30°C. Survival of each strain was monitoring by colony counting.

### Cell adhesion and invasion assay

To assay the adhesion and invasion of the six selected clinical *C*. *albicans* isolates, human endothelial cells (HUVEC) were grown to confluence on 24-well tissue culture plate (Greiner bio-one) in DMEM medium (plus 10% fetal calf serum (FCS), 1% Utra-glutamin and 0.55 ‰ gentamicin sulfate) at 37°C with 5% CO_2_. Then the cells were incubated in FCS free medium for 24 h. After that, HUVEC were labeled by DiO (1:100) (Invitrogen) for 40 min at 37°C. The different clinical *C*. *albicans* strains were cultivated in YPD medium overnight at 30°C. Each *C*. *albicans* strain (1 x 10^6^) was washed by DPBS, and stained with calcofluor (1μg/ml) for 30 min at 37°C. Following washing, *C*. *albicans* were added to DiO labeled HUVEC cells for 2.5 h in FCS free DMEM medium. After extensive washing, cells were detached, interaction of *C*. *albicans* with HUVEC cells were measured by flow cytometry. The infected HUVEC cells were quantified by the fraction of double positive cells (DiO^+^, Calcofluor^+^).

### Western blotting

The different *C*. *albicans* clinical isolates and the SC5314 strain were cultivated in the YPD medium overnight at 30°C. For each strain, the culture supernatant derived from 1x10^6^ cells was collected and treated with Roti-load 1 (Roth), separated by the SDS-PAGE and transferred to a membrane. Levels of secreted Pra1 or Sap1-Sap3 (mainly Sap2) were detected by polyclonal Pra1 or Sap1-Sap3 anti-serum, followed by a HRP-labeled swine anti-rabbit serum as a secondary antibody. The signal was detected by ECL (enhanced chemiluminescence).

### C3b/iC3b surface deposition

The different clinical isolates and *C*. *albicans* strain SC5314 were cultivated in YPD medium overnight at 30°C. For each strain, the culture supernatant derived from 5x10^6^ cells were added to NHS (7.5%). The mixture was further activated by heat inactive *C*. *albicans* pellets in a Mg-EGTA buffer (100 μl) for 30 min at 37°C. After centrifugation, C3b/iC3b surface deposition on *C*. *albicans* pellet were analyzed by flow cytometry using polyclonal goat C3 anti-serum and an Alexa Fluor-488 labeled rabbit anti-goat as a secondary anti-serum.

## Results

### *GPM1* and *PRA1* sequence variations in clinical *C*. *albicans* isolates

**Fig 1:**
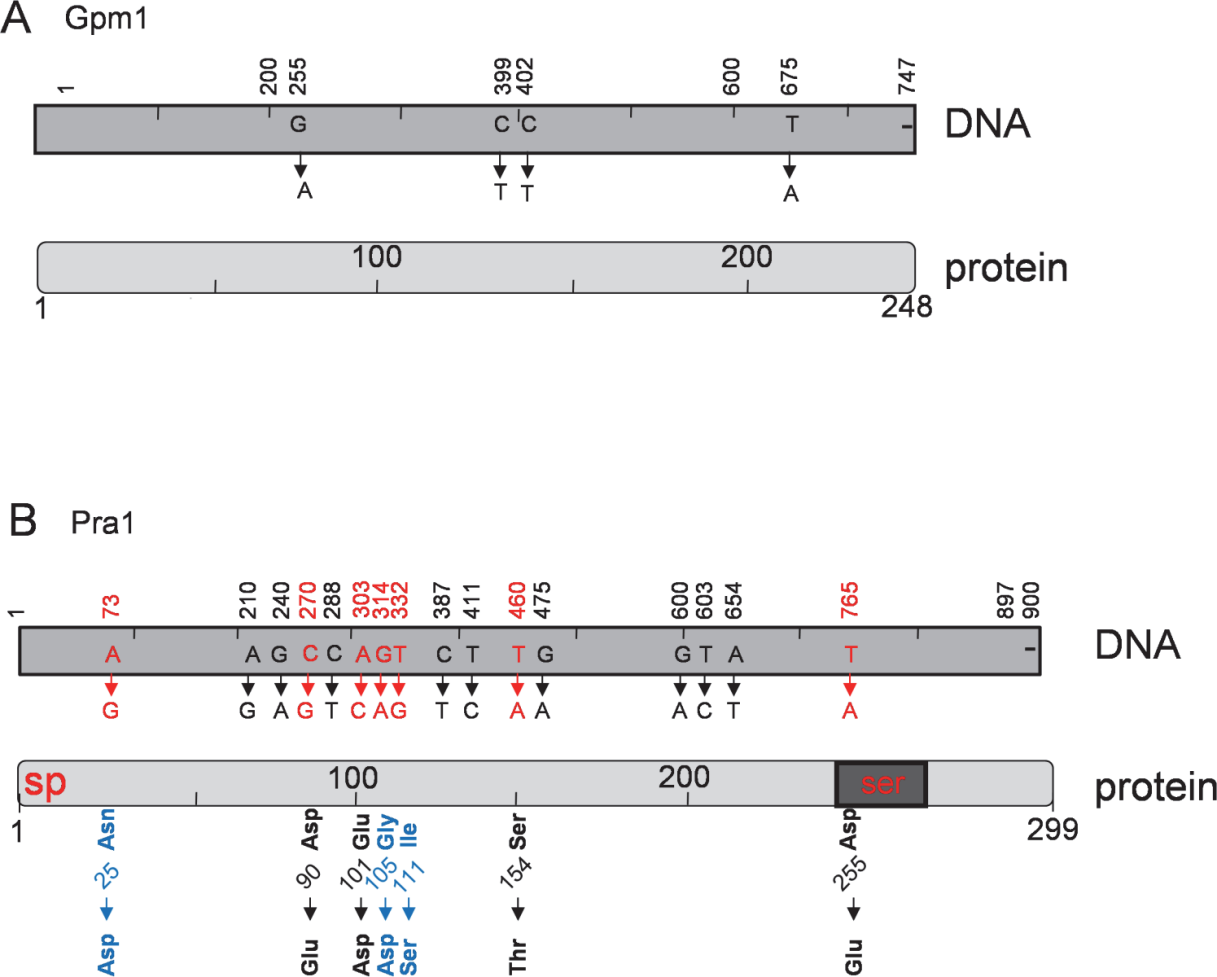
Sequence variations of *GPM1* and *PRA1* in clinical *C*. *albicans* isolates. (**A**) The sequence variations identified in the thirteen clinical *C*. *albicans* isolates are indicated in the structure of the candida *GPM1* cDNA (top) and of the 747-nt Gpm1 protein (bottom). The *GPM1* gene of each clinical *C*. *albicans* isolate was amplified by PCR and the nucleotide sequences were determined. Four synonymous nucleotide exchanges were identified which appeared as homozygous or heterozygous variations and which did not affect the sequence of the Gpm1 protein (lower panel) (see also Table 1). (**B**) Sequence variations identified in the thirteen clinical *C*. *albicans* isolates in the *PRA1* gene and protein. Nucleotide exchanges are indicated for the *PRA1* cDNA (top) and for the 299 amino acid long Pra1 protein (bottom). A total of sixteen nucleotide exchanges occurred as homozygous or heterozygous variation (upper panel). Seven non synonymous nucleotide changes which affect the protein sequence are shown in *red characters*. The structure of the Pra1 protein includes the signal peptide (sp), the serine rich motive (ser), as well as the putative zinc binding region (Zn). Three amino acid exchanges, result in substitution of uncharged residues (i.e. Asn25, Gly105 or Ile111 to negatively charged residues, Asp25, Asp 105 or to a polar uncharged Ser 111 (shown with blue characters). All analyzed clinical isolates had the negatively charged Asp25, in homozygocity. In addition exchange of the non polar Gly_105_ residue to negatively charged Asp residue occurs in some clinical isolates and exchange of the non polar Ile_111_ to the polar, uncharged Ser residue is identified in one single isolate (*marked in blue*).

We analyze sequence variation of two Candida immune evasion genes, *GPM1* and *PRA1* in thirteen clinical *C*. *albicans* isolates, which were collected from infected patients at different infection sites. Genomic DNA of each clinical strain was isolated, the corresponding *GPM1-* and *PRA1* genes were amplified by PCR and the nucleotide sequences were determined. The 747 nt long *GPM1* gene was rather conserved among these clinical isolates. The complete coding region varied at four nucleotide positions, which all result in synonymous exchanges (Fig. 1A and Table 1). The G255A exchange was identified in two isolates as a homozygous exchange and in two other isolates as a heterozygous exchange (Table 1, left panel). The C399T and C402T variations were identified in five strains as homozygous and in two strains as heterozygous exchanges. The T675A variation was present in four strains as homozygous, and in one isolate as heterozygous exchange (Table 1, left panel). Thus *GPM1* has four synonymous nucleotide exchanges with a nucleotide exchange rate of 0.54%, is a relatively conserved fungal gene (Fig. 1).

**Table 1:** Sequence variations of *GPM1* and *PRA1* in clinical *C*. *albicans* isolates.

			GPM1				PRA1															
	Isolate	Infection site	G255	C399	C402	T675	A73	A210	G240	C270	C288	A303	G314	T332	C387	T411	T460	G475	G600	T603	A654	T765
low	# 11	Sacral	GG	CC	CC	AA	GG	AG	GG	CC	CC	AA	GA	TT	CT	TT	TT	GG	GG	TT	AA	AA
	# 3	Oral cavity	no	no	no	no	GG	GG	GG	GG	TT	CC	GG	TT	TT	CC	AA	AA	AA	CC	AA	TT
medium	# 5	Oral cavity	AA	TT	TT	TT	GG	AG	GG	CG	CT	AA	GG	TT	CT	TC	TT	GG	GG	TT	AA	TT
	# 6	Tongue	GG	TT	TT	TT	GG	AA	GG	CC	CC	AA	GG	TT	CT	TT	TT	GG	GG	TT	AA	TT
	# 4	Oral cavity	GG	CT	CT	TT	GG	AG	GG	CG	TT	AC	GG	TT	TT	CC	TT	GG	GG	TT	AA	TT
	# 9	Oral cavity	GG	CC	CC	AA	GG	GG	GG	GG	TT	CC	GG	TT	TT	CC	TT	GG	GG	TT	AA	TT
	# 8	Tracheal secretion	GG	CC	CC	AA	GG	AG	GG	CC	CC	AA	GA	TT	CT	TT	TT	GG	GG	TT	AA	TT
	# 13	Foot	GA	TT	TT	TT	GG	AA	GG	CC	CC	AA	GG	TT	CT	TT	TT	GG	GG	TT	AA	TT
high	# 1	Oral cavity	GG	CC	CC	AA	GG	GG	GG	GG	CC	AA	AA	TT	TT	TT	TT	GG	AA	TT	TT	TT
	# 10	Inguinal	GG	CC	CC	TT	GG	AA	GG	CC	CT	AA	GG	TT	TT	CC	AA	AA	AA	CC	AA	TT
	# 2	Oral cavity	GA	TT	TT	TT	GG	AG	GA	CC	CC	AA	GA	TG	CT	TT	TT	GG	GG	TT	AA	TT
	# 12	Foot	GG	CT	CC	TT	GG	AG	GG	CC	CC	AA	GG	TT	CC	TC	TT	GG	GG	TT	AA	TT
	# 7	Palate	AA	TT	TT	TA	GG	AA	GG	CC	CC	AA	GG	TT	CT	TC	TT	GG	GG	TT	AA	TT
	allele frequency	n	6	12	11	9	26	12	1	8	7	4	4	1	17	11	4	4	6	4	2	2
	strains	hom	2	5	5	4	13	3	0	3	3	2	1	0	5	4	2	2	3	2	1	1
		het	2	2	1	1	0	6	1	2	1	0	2	1	7	3	0	0	0	0	0	0

*GPM1* and *PRA1* genes in each clinical isolate were amplified by PCR and the nucleotide sequences were determined in order to identify gene polymorphisms. Compared to the reference strain SC5314, *GPM1* sequence is rather conserved, among 747-nt long open reading frame, only four nucleotides are exchange either at homozygous (*A*, *highlighted in black*) or at heterozygous scenario (*A*, *highlighted in grey*). As for the *PRA1* gene, among the 900-nt open reading frame, sixteen residues were exchanged either at homozygous (*marked in black*) or at heterozygous scenario (*marked in grey*) with different frequencies. The sequence of the *PRA1* gene was more variable. Within the 900-nt long open reading frame, a total of sixteen nucleotides exchanges were identified (Fig. 1B). Nine nucleotide exchanges presented as synonymous-, and seven as non synonymous exchanges, occurring either in homozygous- or heterozygous setting (Table 1, right panel). Each clinical isolate had at least two nucleotides exchanged among the whole *PRA1* sequence. Substitution of these polar uncharged N-terminally positioned Asn_25,_ Gly_105_ residues to negatively charged acidic Asp_25_ and Asp_105_ residues, or of the non polar hydrophobic Ile_111_ to a polar Ser_111_ residue may be of biological relevance (Fig. 1B). The additional four non synonymous, conservative changes (i.e. Asp90Glu, Glu101Asp, Ser154Thr, as well as Asp225Glu) seem of minor or no significance.

The allelic frequency of the three non-synonymous exchanges in the *PRA1* gene varied in the clinical isolates. All thirteen clinical isolates expressed the Asp25 variant in homozygous setting (Table 1). The Gly105Asp exchange was rare, one isolate had Asp105 as a homozygous, and in three isolates as a heterozygous variant. The Ile111Ser exchange appeared as heterozygous variation in one single isolate (Table 1). The *PRA1* gene had an overall nucleotide exchange rate of 1.78%, with nine synonymous and seven non synonymous exchanges. Thus, *GPM1* and *PRA1* represent conserved fungal immune evasion genes.

### Surface expression levels of Gpm1 and Pra1 in the clinical *C*. *albicans* isolates

**Fig 2:**
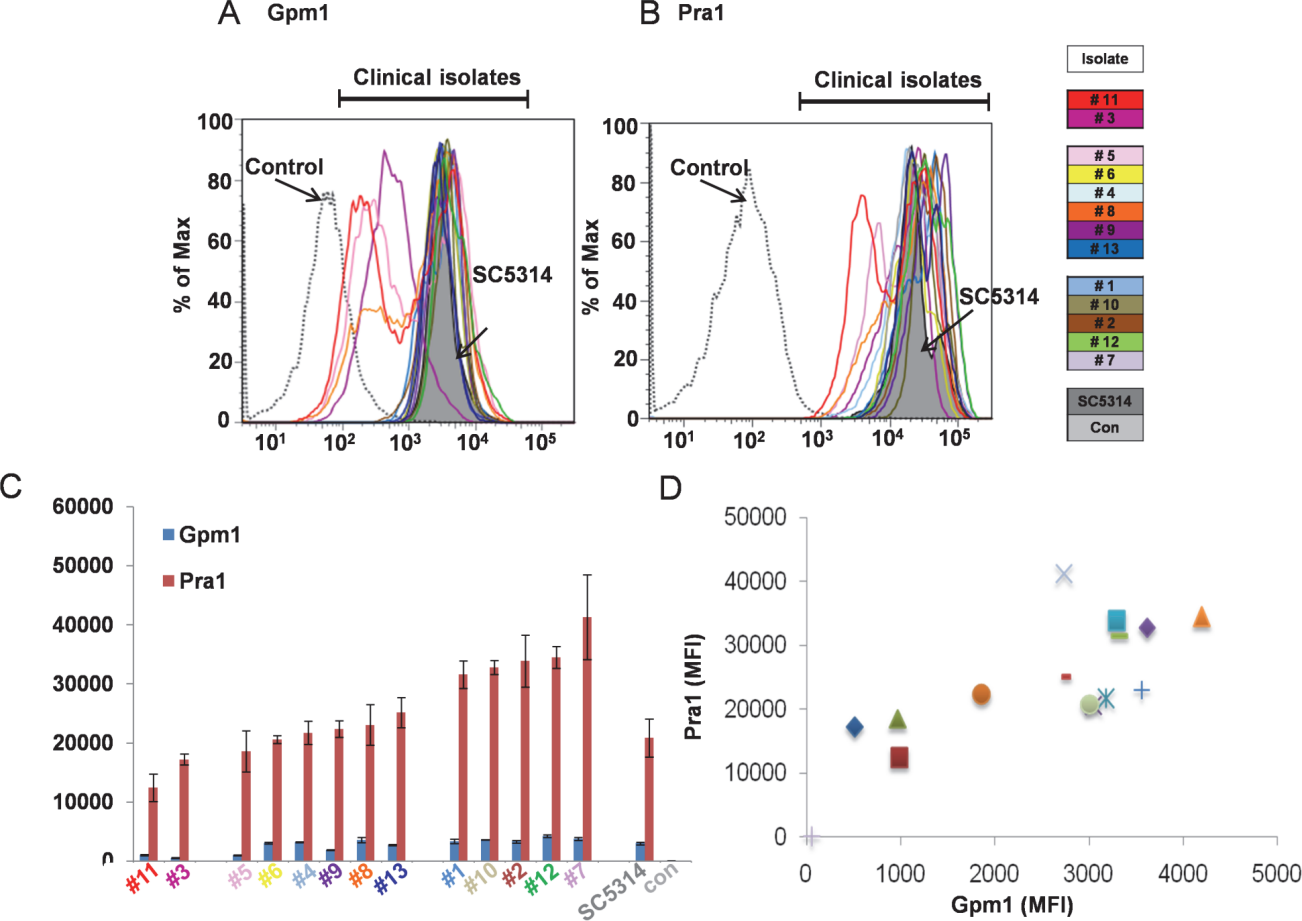
Gpm1 and Pra1 expression levels vary among the *C*. *albicans* isolates. Gpm1 (**A**) and Pra1 (**B**) surface expression of the thirteen clinical *C*. *albicans* isolates. Each *C*. *albicans* isolate as well as the reference strain SC5314 was incubated in Gpm1- or Pra1 specific anti-serum and after washing bound antibodies were detected by flow cytometry using secondary Alexa Fluor-488, or Alexa Fluor-647 labeled goat-anti rabbit serum. Control means that SC5314 was incubated with pre-immune serum instead of anti Pra1 or anti Gpm1 sera. Each panel shows representative results of three independent experiments. (**C**) Median fluorescence intensity of Gpm1 and Pra1 from three independent experiments. The data shown represent mean values ± SD of three separate experiments. (**D**) Correlation of Gpm1- and Pra1 surface levels for the in thirteen clinical isolates and SC5314. When further analyze the expression level of Candida Gpm1 and Pra1 within these thirteen clinical isolates, we found that Gpm1 and Pra1 surface levels correlated with each other. Most of the high Gpm1 expressing Candida isolates had relatively high Pra1 surface levels (Fig. 2D). Similarly the medium or low Gpm1 expressing strains had medium/low Pra1 surface levels, respectively. The exceptions were isolates J#5 and J#9, of which both had low or high Gpm1, but medium Pra1 levels (Table 2). Thus Gpm1 and Pra1 surface levels are variable among the clinical isolate and surface expression levels of the two fungal immune evasion proteins correlate with each other to a great extent.

To identify additional variables relevant for fungal immune fitness, we next analyzed surface expression levels of the two immune escape proteins. Gpm1 expression levels varied among in the clinical strains and ranged from 512 to 4200 (MFI). Pra1 expression levels ranged from 12393 (J#11) to 41282 (J#7) (MFI) (Fig. 2A-2C). Thus Gpm1 surface levels varied by a factor of 8.2 and that of Pra1 by 3.3.

**Table 2:** Gpm1 and Pra1 surface expression levels.

	Isolate	Gpm1	Pra1
		(Mfi)	(Mfi)
Low	# 11	987	12393
	# 3	512	17197
Medium	# 5	968	18597
	# 6	3051	20577
	# 4	3181	21693
	# 8	1864	22341
	# 9	3558	23043
	# 13	2714	25119
High	# 1	3317	31564
	# 10	3612	32778
	# 2	3292	33854
	# 12	4194	34493
	# 7	2734	41282
	SC5314	2998	20844
	Control	54	72
		Gpm1	Pra1
	Low	< 1700	<17500
	Medium	1701–3200	17501–30000
	High	> 3200	> 30000

The median fluorescence intensities for both proteins’ expression level are shown in. Due to different surface expression level of both proteins, these thirteen strains were divided into three groups: low (Marked in light grey), medium (marked in dark grey) and high (marked in black). Six clinical isolates (two from the low group, two from the medium group and another two from the high group according to Pra1 surface expression) were selected for further function analysis. MFI (mean fluorescence intensity) Gpm1 and Pra1 bind several human innate immune regulators. Gpm1 binds human Factor H and plasminogen, but not C4BP. Pra1 binds Factor H, C4BP and plasminogen [25,35]. In order to define whether Gpd2 and Pra1 surface levels affect fungal immune fitness, firstly the clinical strains were grouped as low (MFI of Pra1< 17500, two isolates), medium (MFI of Pra117501-30000, six isolates) and high expressing strains (MFI of Pra1>30000, five isolates) (Table 2) according to their Gpm1/Pra1 surface levels. Two strains were selected from each group and tested for (i) regulator binding, (ii) survival in complement active, Factor H depleted human serum, (iii) adhesion to human endothelial cells, as well as (iv) for their effect on complement activation.

### Binding of the human immune regulators Factor H, C4BP and plasminogen to clinical *C*. *albicans* isolates

**Fig 3:**
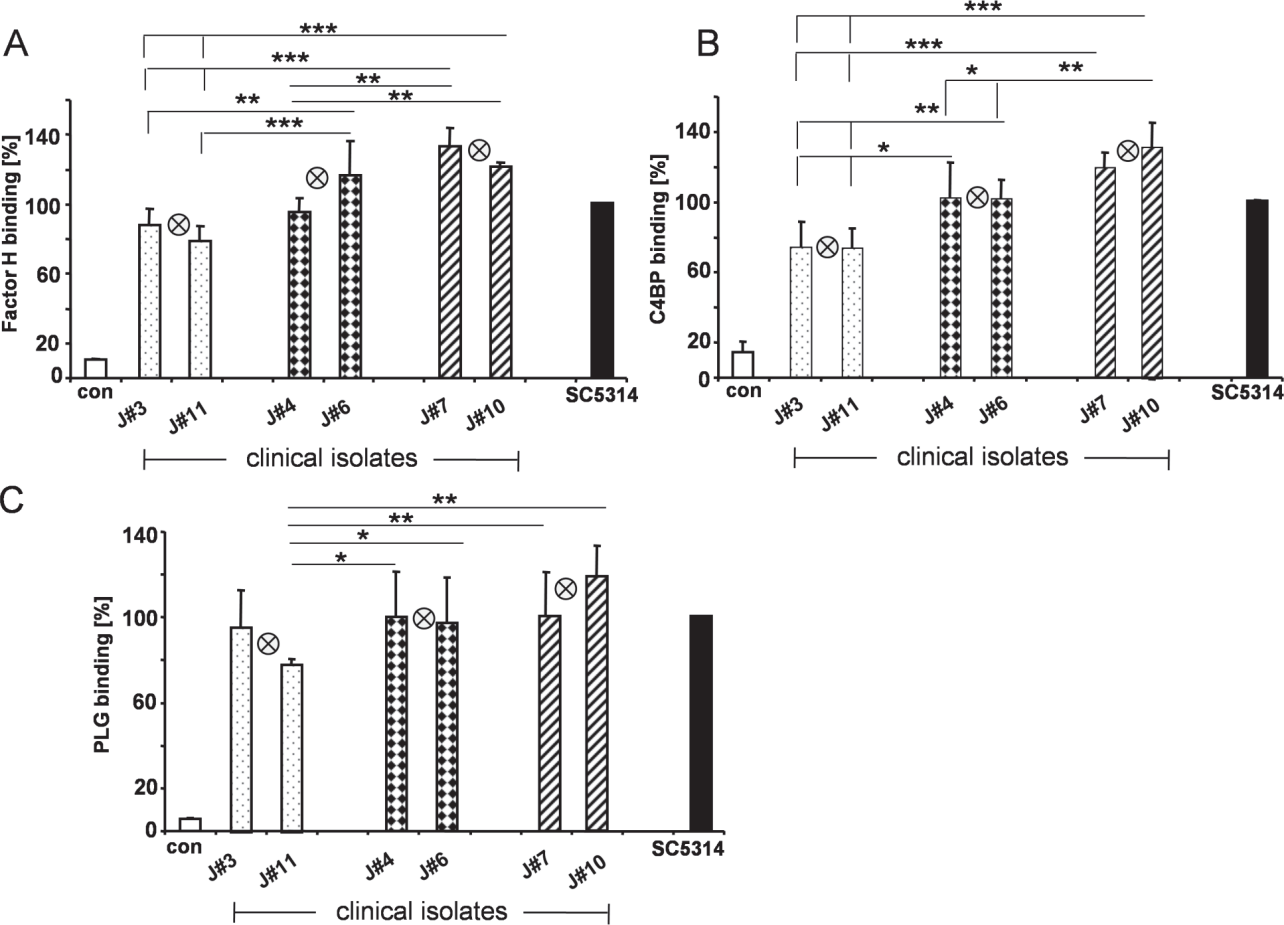
Binding of the human complement regulators to selected *C*. *albicans* isolates. The selected low Gpm1- and Pra1 expressing yeast isolates (J#3 & J#11), the two medium (J#4 & J#6), as well as two high expressing strains (J#7 & J#10) and SC5314 were incubated in NHS. After washing surface bound Factor H- (**A**), C4BP- (**B**), as well as plasminogen (**C**) was detected with the corresponding anti serum in combination with secondary labeled Alexa Fluor-647 rabbit anti-goat, or goat anti-rabbit serum. Bound immune regulators were quantified by flow cytometry. The mean values of each group, i.e. the low, medium or high Gpm1/Pra1 expressing isolates are indicated by the crossed circle in the middle of each group. The data shown represent mean values ± SD of three separate experiments. Statistical significance of differences was determined using Student's *t*-test. *, *p*≤0.05; **, *p*≤0.01; ***, *p*≤0.001.Similarly, C4BP binding varied. This classical pathway regulator bound with moderate intensity to the low expressing isolates J#3 and J#11, i.e. 75% and 74%, with increased levels to the medium expressing strains, i.e. 102% and 102%, with highest intensity to the two high expressing strains, i.e. 119% and 132%. Both high expressing fungal isolates bound ca. 50% more C4BP to their surface as compared to the low expressing isolates (Fig. 3B).

To defined how Gpm1- and Pra1 surface levels contribute to regulator attachment, the selected low (J#3, J#11), medium (J#4, J#6) and high expressing isolates (J#7, J#10) were challenged with NHS and after washing attached Factor H, C4BP as well as plasminogen was evaluated by flow cytometry. Each strain bound all three human plasma regulators, however the regulators bound with different intensity. To allow a direct comparison, regulator binding to the reference strain SC5314 was set to 100%. Factor H bound to the low expressing isolates, J#3 & J#11 with low (i.e. 88%, 79%), to the medium expressing strains, Jn#4 & J#6 with medium (i.e. 96%, 117%), and to the high expressing isolates, J#7 & J#10, with high intensity (i.e. 133%, 122%). Thus surface attachment of the human complement regulator Factor H varied by ca. 44% and correlated with Gpm1-/Pra1 surface levels (Fig. 3A).

In addition, also plasminogen binding varied. Plasminogen bound with moderate intensity to the low expressing strains, i.e. 96% and 78%, with higher intensity to the medium strains, i.e. 100% and 97% and with strongest intensity to the high expressing strains i.e. 101% and 119%. The difference between the high and low expression strain is ca. 23% (Fig. 3C). Thus the tested three human complement regulators bound with different intensity to the clinical C. albicans isolates and the intensity of regulator binding correlated with Gpm1- and Pra1 surface levels.

### Immune fitness of the *C*. *albicans* isolates

**Fig 4:**
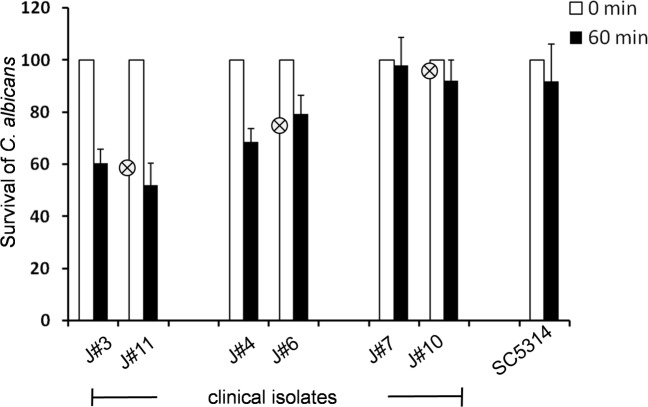
Survival of the selected clinical isolates in complement active, Factor H depleted human serum. The selected clinical *C*. *albicans* isolates and the reference strain SC5314 strain were cultivated in YPD medium overnight at 30°C. Then the yeast cells (1x10^4^/ml) were incubated in complement active, Factor H depleted human serum (10%) for 60 min at 37°C. At start (time 0) and after 60 min incubation, aliquots were removed and the number of live yeast cells was determined by plating on the YPD agar plate, which then were cultivated for 2 days at 30°C. Survival yeast number at time 0 was set 100%. The mean values of each group, i.e. the low, medium or high Gpm1/Pra1 expressing isolates are indicated by the crossed circle in the middle of each group. Data shown represent the mean values ± SD of three separate independent experiments. Pra1 surface levels affect fungal adhesion and invasion to human endothelial cells.

In order to define how Gpm1 and Pra1 surface levels and regulator attachment contribute to fungal immune fitness, the clinical *C*. *albicans* isolates were first challenged with normal human serum and after incubation the cell survival was evaluated. The number of *C*. *albicans* cells at time zero was set 100%. All clinical isolates survived in NHS and no significant differences were detected (data not shown). We interpreted that Factor H as the central complement inhibitor when attached to the fungal surface protects Candida from complement damage. To further compare the immune fitness of the various fungal isolates in the absence of Factor H, and their survival in Factor H depleted complement active human serum were analyzed, both low expressing strains showed relative poor survival, as 61% and 52% of the yeast cells were recovered. The medium expressing isolates, had a good (69% and 79%) and both high expressing isolates had an even better survival (98% and 92%) (Fig. 4). The difference in survival between high and low expressing isolates corresponds to ca. 40%. These data demonstrate that the Factor H acquired by the fungal pathogens contributes to a significant extend for immune escape and that addition role of Pra1 and Gpm1 control complement attack.

**Fig 5:**
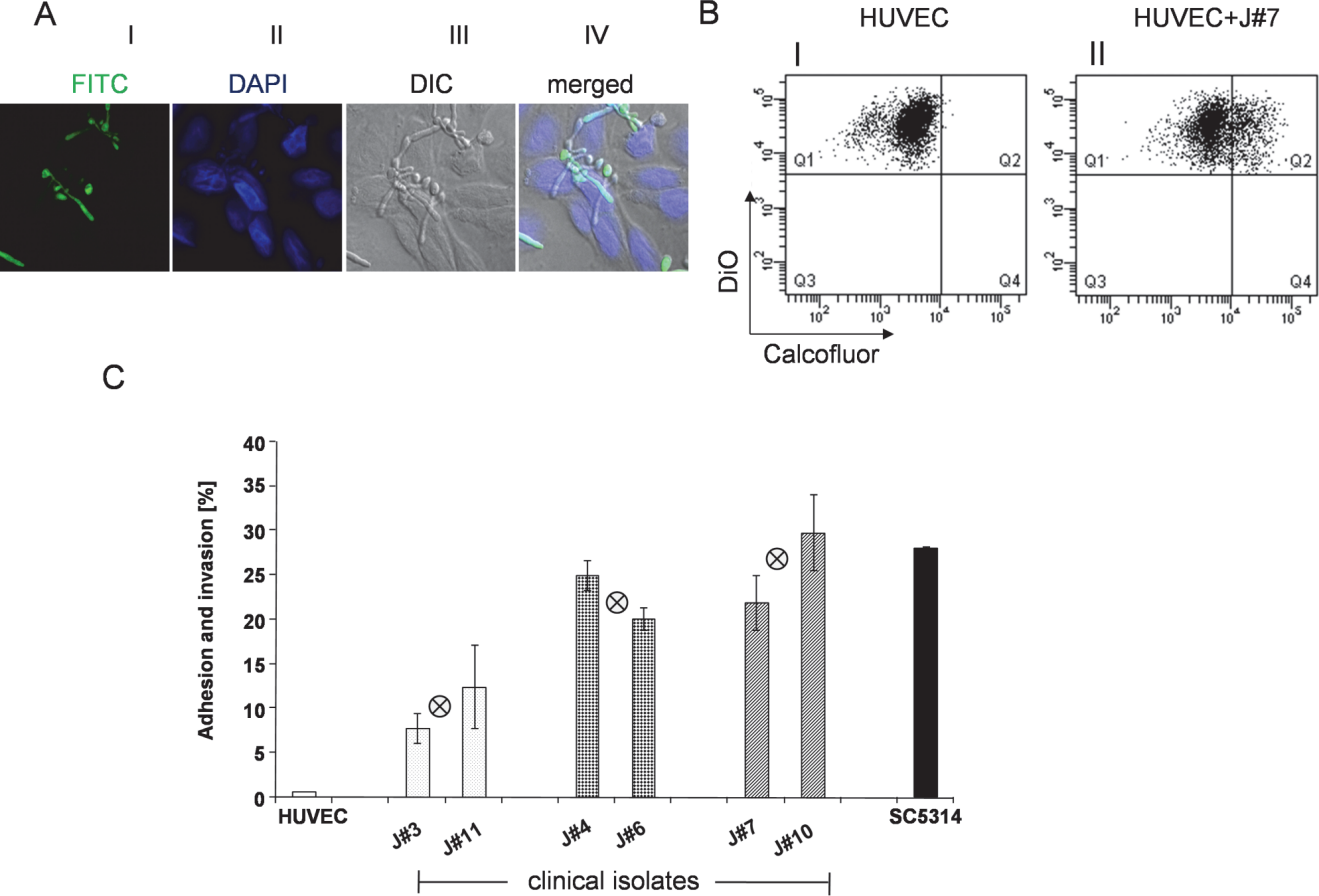
The clinical *C*. *albicans* isolates attach to human endothelial cells. (**A**) J#7, the high Gpm1-, Pra1 expressing clinical isolate was co-cultivated with human endothelial cells (HUVEC) for 2.5 h at 37°C. After washing, yeast cells were stained by anti-cell wall serum and in combination with FITC labeled goat anti rabbit serum (green fluorescence). HUVEC cells were stained by DAPI (blue) and evaluated by laser scanning microscopy. *C*. *albicans* (green) either adhered or invaded the HUVEC cells (blue). (**B**) To quantify how Gpm1- and Pra1 surface levels influence the interaction of different clinical *C*. *albicans* isolates with HUVEC, overnight cultures of the selected clinical isolates and of the reference strain SC5314 (1 x 10^6^) were washed in DPBS, then stained with calcofluor (1 μg/ml) for 30 min at 37°C. After washing the yeast cells were added to DiO (1:100) labeled HUVEC cells, which were kept in FCS (fetal calf serum) free DMEM medium. After co-cultivation (2.5 h), unbound Candida cells were removed by extensive washing, then HUVEC cells together with the adherent and invaded *C*. *albicans* cells were detached, washed again and the samples were evaluated by flow cytometry. The blot shown here is an example of one clinical isolate (J#7). DiO labeled HUVEC cells were identified as single positive cells (DiO^+^, Calcofluor^-^) *(panel I)*. HUVEC cells with adherent and ingested yeast cells were identified as double positive cells (DiO^+^, Calcofluor^+^) (*panel II*). (**C**) Comparison of the infection ability of different clinical *C*. *albicans* isolates. Different clinical *C*. *abicans* isolates, reference strain SC5314 and HUVEC cells were prepared and labeled as shown in (B). After co-cultivation (2.5 h), the percentage of infected HUVEC cells by different clinical *C*. *albicans* isolates were recorded by flow cytometry as double positive cells. The percentage of double positive HUVEC cells were used to indicate the infection ability of different *C*. *albicans* isolates. The mean values of each group, i.e. the low, medium or high Gpm1/Pra1 expressing isolates are indicated by the crossed circle in the middle of each group. The bars represent the mean values of three independent experiments ± S.D. To better quantify the adhesion and invasion ability of different clinical strains to human endothelial HUVEC cells, *C*. *albicans* isolates labeled with calcofluor were co-cultivated with DiO labeled HUVEC cells, afterwards the interaction of yeast and human cells were analyzed by flow cytometry. Unbound and loosely attached Candida cells were removed by washing. HUVEC cells that had no *C*. *albicans* attached were identified as single positive cells (Calcofluor^-^, DiO^+^) (Fig. 5 *B*, panel I). In contrast HUVEC cells with adherent and invaded Candida cells were identified as double positive cells (Calcofluor^+^ and DiO^+^) (Fig. 5*B*, panel II). Upon co-cultivation of the high expressing strain J#7, about 23% of the human endothelial cells had *C*. *albicans* infected.

Both adhesion to and invasion into human endothelial cells are critical for fungal survival in the infected host and for pathogenicity [29,45,46]. To monitor fungal adhesion/invasion to human endothelial cells the clinical isolate J#7 was co-cultivated with human endothelial cells (HUVEC). After washing, the Candida cells were stained with a cell-wall reacting anti-serum and the human cells with DAPI and the cells were analyzed by laser scanning microscopy (Fig. 5A). Upon incubation Candida yeast cells adhered to the human cells, hyphae formation was observed and Candida hyphae invaded into the human endothelial cells.

This approach allowed to compare infection of the various fungal isolates to human endothelial cells and to assay whether Gpm1/Pra1 surface levels affect interaction with human endothelial HUVEC cells. The six selected clinical isolates showed different rates of adhesion and invasion. Both low expression strains (J#3 and J#11) had relatively weak infection ability with 8 or 13% of the human cells appearing as double positive cells. The medium expressing isolates (J#4 and J#6) bound to 25 and 20% of the human cells and the high expressing isolates (J#7 and J#10) interacted almost better, i.e. 22 and 30%. Thus the two high expressing clinical *C*. *albicans* isolates were more efficient in contacting human endothelial cells by about 60% than the low expressing isolates, indicating that the fungal surface proteins Gpm1 and Pra1 are relevant for contact with human cells and Gpm1- Pra1 surface levels contribute to fungal interaction with human endothelial cells.

### Secretion levels of Pra1

**Fig 6:**
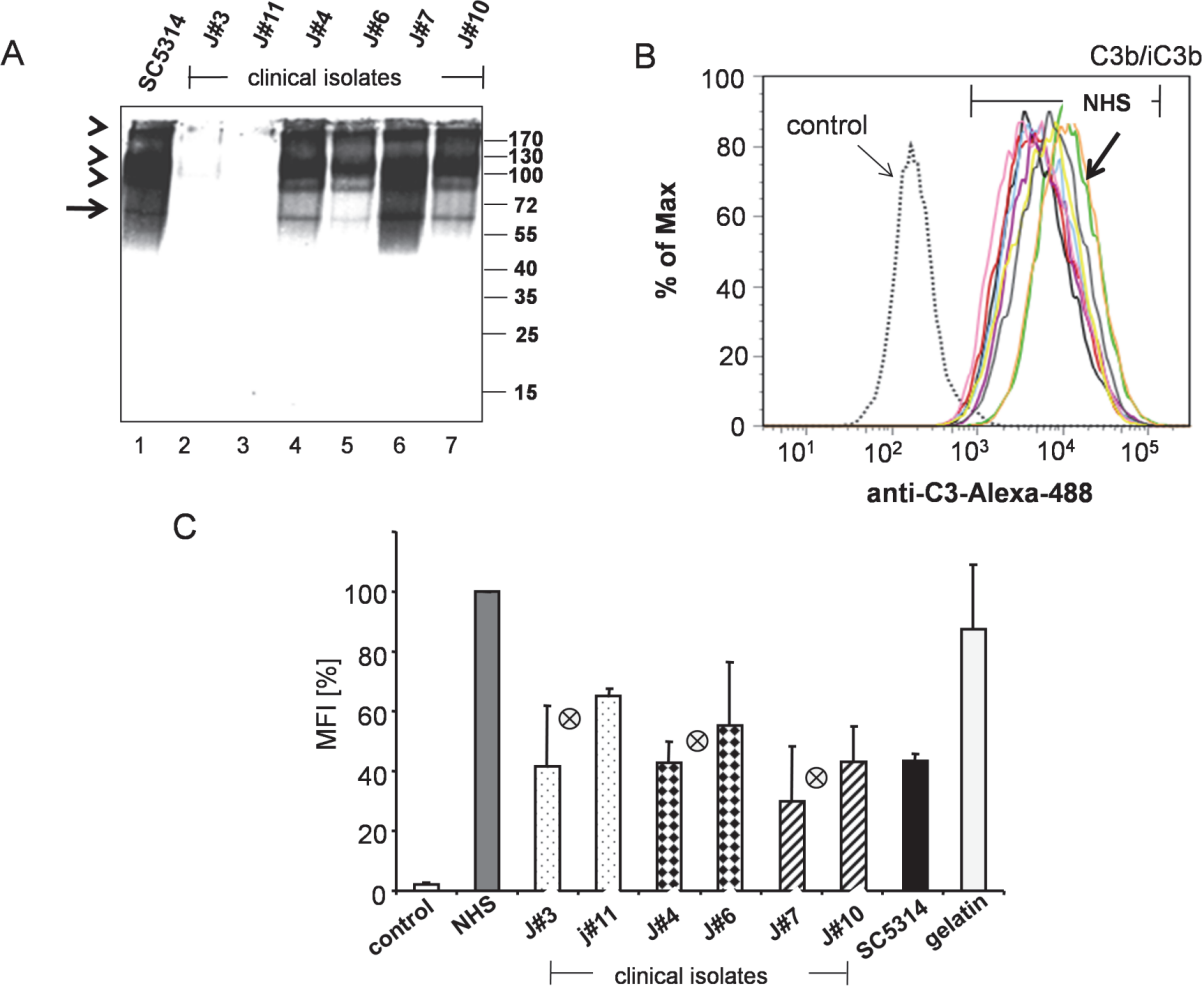
Pra1 secretion and effect of secreted Pra1 on C3b/iC3b surface deposition. (**A**) Presence of Pra1 in the culture supernatant. Culture supernatant (YPD medium following overnight culture) of the selected clinical *C*. *albicans* strains (1x10^6^ cells) was separated by SDS-PAGE, transferred to a membrane and Pra1 levels were detected by rabbit Pra1 antiserum, followed by a HPR swine anti-rabbit serum as a secondary antibody. Pra1 was detected as a 60 kDa protein in culture supernatant derived from medium (lanes 3 and 4) and of high expression Gpm1/Pra1 strains (lanes 6 and 7). The additional bands of higher molecular mass and of slower mobility (arrow heads), represent Pra1 in complex with additional soluble proteins. Pra1 was not detectable in the culture supernatant derived from the two low Gpm1/Pra1 expressing strains (lanes 2 and 3). The arrow shows the 60 kDa Pra1 monomer and the position of the additional dimmer or multimeric forms and Pra1 containing complexes are likely indicated by the arrow heads. (**B**) Supernatant derived from the clinical *C*. *albicans* isolates blocked C3b/iC3b deposition on the yeast surface. The selected *C*. *albicans* clinical isolates were cultivated in YPD medium overnight, and culture supernatant derived from 5x10^6^ cells of each isolate was added to NHS (7.5%) diluted in Mg-EGTA, then heat treated C. *albicans* were challenged with this serum-supernatant mixture for 30 min at 37°C. Following washing C3b/iC3b surface deposition was analyzed by flow cytometry using goat anti C3 serum. Candida cells treated in the absence of NHS are shown as control. The histogram shown here is one representative experiment out of three performed. (**C**) The mean values of median fluorescence intensity of C3b/iC3b from three independent experiments. C3B/iC3b levels on the surface of cells incubated in NHS were set 100%. The mean values of each group, i.e. the low, medium or high Gpm1/Pra1 expressing isolates are indicated by the crossed circle in the middle of each group. The mean values of median fluorescence intensity from three independent experiments ± SD are shown.

Surface levels of Gpm1 and Pra1 varied among the clinical isolates. In addition Pra1, but not Gpm1, is secreted into the culture medium [34]. Therefore we analyzed and compared Pra1 levels in the culture medium. Culture supernatant was collected after 24 h, separated by SDS-PAGE, transferred to a membrane and Pra1 was identified by Western blotting. Pra1 was detected as a 60 kDa band in the supernatant of the medium- and the high expressing isolates, as well as in culture supernatant of the reference strain SC5314 (Fig. 6*A*). Pra1 was not detectable in supernatant collected from both low expressing isolates, J#3 and J#11 (Fig. 6A, lanes 3 and 2). Thus the medium and high, but not the low expressing Pra1 expression strains have detectable levels of Pra1 in the culture supernatant. Released Pra1 level correlates with the expression level at the yeast surface. The band with mobility of around 60kDa is the Pra1 protein, the upper binds are likely the dimeric or multimeric variant of Pra1, or even complexes of Pra1 with other Candida proteins.

In order to define whether Pra1 levels in the supernatant correlate with that of other fungal virulence factor, secreted aspartyl proteases 1–3 (mainly Sap2) level were evaluated in by Western blotting [47,48]. The low Pra1 expressing strain J#3, but not isolate J#11, one high Pra1 expressing isolateJ#10 and the reference strain SC5314 had no detectable levels of Sap2 in YPD-culture supernatant. Sap2 was released by both medium-, one low- and one high Pra1 expressing strain isolates (S1 Fig.). Thus Sap2 release varied among the isolates and did not correlate with Pra1 levels.

### Secreted Pra1 affects C3b/iC3b surface deposition

Soluble Pra1 inhibits C3 activation and C3 processing and contributes to fungal survival [34]. Given the different Pra1 levels in the culture supernatant of the tested clinical strains, we next asked whether Pra1 secreted into the culture supernatant influences C3b/iC3b deposition on the fungal surface. To this end, culture supernatant derived from the low, medium and high expressing *C*. *albicans* isolates was first added to NHS and then used to challenge heat treated *C*. *albican*s. Following incubation and extensive washing, C3b/iC3b deposition on the fungal surfaces was evaluated by flow cytometry. In this case the median fluorescent intensities of surface deposited C3b/iC3b varied. Supernatant derived from the two low Pra1 secreting isolates, resulted in high C3b deposition. Supernatant derived from the medium and high Pra1 secreting isolates caused lower C3b surface deposition (Table 3). C3b/iC3b surface level challenged with NHS in the absence of the *C*. *albicans* supernatant was set as 100% (i.e. MFI 10946) (Fig. 6B, *green curve*). C3b/iC3b surface levels upon treatment with supernatant derived from the low expressing isolates (J#3 and J#11) was reduced by 58% and 35%, for the medium expressing isolates (J#4 and J#6) by 57% and 45%, and for the high expressing strains (J#7 and J#10) by 70% and 57%, respectively (Fig. 6C). High Pra1 levels blocked C3b/iC3b surface deposition by about 27% better, compared to the low Pra1 expressing strain. Thus Pra1 levels can be considered as a biomarker on complement control as evidenced by C3b/iC3b surface deposition onto the fungal surface.

**Table 3:** Mean fluorescence intensity of the C3b/iC3b surface deposition.

Isolate	iC3b	
	(Mfi)	
# 11	7290	low
# 3	6116	low
# 6	4397	medium
# 4	5239	medium
# 10	5624	high
# 7	4678	high
SC5314	4552	
Control	172	
serum	10947	
Gelatine	11235	

The mean fluorescence intensities of the C3b/iC3b surface deposition from one experiment shown in Fig. 6B are shown in Table 3. Due to different surface expression level of Gpm1/Pra1, six clinical isolates (J#3 and J#11, J#4 and J#6 as well as J#7 and J#10) show different ability on complement control as indicated by different surface C3b/iC3b deposition.

## Discussion

Microbial pathogens modulate and control the innate immune responses in order to survive in an immunocompetent host. Pra1 and Gpm1 are two central fungal complement evasion proteins of *C*. *albicans*. Here we analyzed sequence variation, as well as expression levels of these two fungal immune evasion proteins among clinical *C*. *albicans* isolates. The nucleotide sequence revealed *GPM1* and *PRA1 as* conserved fungal immune evasion genes. Both immune evasion proteins were expressed with different levels at the fungal surface. In addition, Pra1 secretion levels varied. The surface levels of Gpm1 and Pra1 correlated with each other. The level of these two immune evasion proteins influenced: (i) fungal binding of the human plasma regulators, Factor H, C4BP and plasminogen, (ii) fungal survival in complement active, Factor H depleted human serum, (iii) fungal adhesion to human endothelial cells, and (iv) also C3b/iC3b deposition onto the fungal surface.

Candida *GPM1* and *PRA1* represent rather conserved fungal genes, have little sequence variation and show gene variation rates of 0.54 and 1.78% in the thirteen clinical *C*. *albicans* isolates tested. The allelic frequencies of the four synonymous nucleotide exchanges within the *GPM1* gene ranged from 0.31 to 0.50. The allelic frequency for each of the sixteen exchanges of the *PRA1* gene ranged from 0.08 to 1.0. The *PRA1* gene had seven non synonymous exchanges, and four of these exchanges cause a conservative amino acid replacement. The three likely more relevant exchanges at positions 25, 105 and 111 were represented with allelic frequencies of 1.0, 0.23 and 0.07, respectively. The two exchanges at position 25 and 105, i.e. Asn25 and Gly105, replace a neutral amino acid a charged and acidic Asp residue. In addition Ile111 is changed to a hydrophobic Ser residue (Fig. 1). All tested clinical isolates and the tested strain SC5314 had nucleotide G73, resulting in Asp25. Genbank reports for strain SC5314 at this position two different nucleotides. The initial sequence reports A73 (accession no. U84261), coding for Asn25 [49], and also the variant G73 (Asp25) is reported [50].

Candida Gpm1 and Pra1, like other pathogen derived complement evasion proteins bind many human plasma proteins, and attach several of the host immune regulators. In general terms complement evasion proteins of pathogenic microbes present two major classes: The first group includes immune evasion proteins which have a conserved sequence repertoire, and these proteins show no or rather little sequence diversity. Proteins of this conserved group, include Gpm1 and Pra1, the two fungal proteins studied here, the CRASPs proteins of *Borrelia* species and also the fHbp protein of group B *N*. *meningitidis* isolates [42]. However group II complement evasion proteins, have a high degree of sequence diversity and a modular structure, but maintain conserved binding characteristics for human complement regulators and for host proteins. Examples for such highly polymorphic microbial proteins are, the M proteins family of *S*. *pyogenes* and the PspC protein family of *S*. *pneumonia* [38,39,40,41].

At present the regulator binding regions within Gpm1 and Pra1 have neither been localized, nor mapped to certain protein regions. The *PRA1* sequence includes an N-terminal cysteine-rich domain, an internal collagen-like domain, a zinc binding region and a non collagen type region within C-terminus [49,51]. The collagen-like domain has been proposed to be relevant for anchoring, attaching and colonization of the fungus to human extra-cellular matrices.

Expression levels of fungal complement evasion proteins vary among the isolates but correlate with regulator attachment and with survival in human Factor H depleted serum. The clinical *C*. *albicans* isolates with high Gpm1/Pra1 levels bound the protective human regulators more efficiently. The high expressing isolates bound about 50% more Factor H and C4BP to their surface than the low expressing isolates. Plasminogen binding was also increased by 23% (Fig. 3). Upon challenge with normal human serum, all tested *C*. *albicans* survived and no significant difference was detected. Thus indicating that surface attached Factor H, although with different levels efficiently protected these isolates from alternative complement attack [17,52]. However, the high expressing clinical *C*. *albicans* isolates control the complement challenge more efficiently and survived better upon challenge by human Factor H depleted serum, i.e. 98 and 92.1%. Both low expression strains were more susceptible to this serum challenge and in this case only 60.5 or 52% of the cells survived (Fig. 4). Thus Gpm1 and Pra1, likely in combination with other fungal virulence factors contribute to fungal immune fitness and to fungal survival.

*C*. *albicans* as a tissue residing pathogen not only needs to block the complement barrier, but also to cross and to penetrate endothelial- as well as epithelial cell layers. Thus after crossing the complement barrier, the next steps critical for fungal infection are adhesion and invasion to human endothelial cells, breaching the endothelial barrier and dissemination into deeper tissue layers [45]. The analyzed clinical isolates showed a different activity for endothelial cell adhesion and invasion and this difference is influenced and modulated by Gpm1 and Pra1 surface levels. The high expressing Candida isolates showed about 60% better infection to the human endothelial cells, as compared to the low expressing isolates.

Pra1 levels in the culture supernatant varied among the clinical strains and influenced C3b/iC3b surface deposition. Apparently Pra1 is not the only fungal C3 inhibitor. Supernatant of isolates J#3 and J#11, that both lacked detectable Pra1 levels also blocked C3b/iC3b surface opsonization (Fig. 6). Such additional fungal C3 inhibitory or degrading proteins include Sap2, which as shown here is however differently regulated from Pra1. *C*. *albicans* uses a set of virulence factors or virulence determinants to control and to modulate host complement attack. During the various infection stages the individual virulence determinants control fungal immune evasion, adjust cell adherence and tissue interaction (Fig. 6).

*C*. *albicans*, similar to many other Gram positive or Gram negative pathogenic bacteria expresses several Factor H-, C4BP- and/or plasminogen binding evasion proteins. At present, four Factor H (Gpm1, Pra1, Hgt1p, Gpd2), two C4BP (Pra1 and Hgt1p), and ten plasminogen binding proteins (Gpm1, Pra1, Gpd2, alcohol dehydrogenase, thioredoxin peroxidase, catalase, transcription elongation factor, glyceraldehyde-3-phosphate dehydrogenase, phosphoglycerate kinase and fructose bisphosphate aldolase) have been identified for *C*. *albicans*. Such an acquisition of multiple host complement regulators and also of other human immune effector proteins is a common and important escape strategy that is used by many, likely even by all pathogenic microbes. The levels of surface attached host regulators, Factor H, C4BP and plasminogen by different clinical isolates correlated in a positive manner, with Gpm1- and Pra1 expression levels at the fungal surface. Although a clear correlation existed, there are also some variations, showing that additional proteins and factors contribute to fungal immune evasion.

Taken together, *GPM1* and *PRA1* of *C*. *albicans*, are rather conserved fungal immune evasion genes which are expressed at different levels among the tested clinical isolates, influencing fungal immune fitness. These fungal evasion proteins influence many steps of the infection process, in particular acquisition of human complement regulators, survival of complement challenge by active, Factor H depleted human serum, adhesion and invasion to human endothelial cells, as well as blockade of surface C3b/iC3b deposition. *C*. *albicans* expresses several complement and immune escape proteins at the same time, explaining why a targeting or an inhibiting approach of a single gene does not cause substantial changes in the immune response. Therefore approaches that aim to direct and inactivate all fungal escape proteins at the same time seem more appropriate.

## Supporting Information

S1 Fig Levels of Candida Sap1/Sap2/Sap3 in the culture supernatant of the selected clinical *C*. *albicans* strains. The selected *C*. *albicans* clinical strains and of the reference SC5314 strain were cultivated in YPD medium overnight at 30°C. Culture supernatant derived from 1x10^6^ cells of each *C*. *albicans* strain was separated by SDS-PAGE and transferred to a membrane and developed with a polyclonal rabbit anti Sap1/Sap2/Sap3 serum, followed by a HPR swine anti-rabbit serum as a secondary antibody. The 42 kDa Sap2 band was detected in culture supernatant derived from one low, two medium and one high Gpm1/Pra1 expressing isolate. The figure shows a representative experiment out of three performed.
(DOC)
