# Supplementary material for: Sequence Variations and Protein Expression Levels of the Two Immune Evasion Proteins Gpm1 and Pra1 Influence Virulence of Clinical Candida albicans Isolates
Source: PLoS One. 2015 Feb 18;10(2):e0113192. doi: 10.1371/journal.pone.0113192 (PMC4334649; doi:10.1371/journal.pone.0113192)

**Supporting Information**

**Sequence Variations and Protein Expression Levels of the two Immune Evasion Proteins Gpm1 and Pra1 Influence Virulence of Clinical *Candida albicans* Isolates**

**Shanshan Luo^1^,**^$^**, Uta-Christina Hipler^2^, Christin Münzberg^1^, Christine Skerka^1^, Peter F. Zipfel*^1,3^**

From ^1^ Department of Infection Biology, Leibniz-Institute for Natural Product Research and Infection Biology, Hans-Knöll-Institute, Jena, Germany; ^2^Friedrich-Schiller-University Hospital, Clinic of Dermatology and Allergology; ^3^Friedrich-Schiller-University, Jena, Germany.

^$^Current address, Institut für Medizinische Mikrobiologie, Immunologie und Hygiene, Technische Universität München.

*Address correspondence to: Peter F. Zipfel, Department of Infection Biology, Leibniz Institute for Natural Products Research and Infection Biology, Hans-Knöll Institute, Beutenberg str. 11a, 07745 Jena, Germany, Phone: +49 (0) 3641 532-1300, Fax: +49 (0) 3641 532-0807; E-mail: [peter.zipfel@hki-jena.de](mailto:peter.zipfel@hki-jena.de)

**Supporting Information Legends**

Supplementary Figure 1: Levels of Candida Sap1, Sap2 and Sap3 in the culture supernatant of the selected clinical *C. albicans* strains. The selected *C. albicans* clinical strains and of the reference SC5314 strain were cultivated in YPD medium overnight at 30 °C. Culture supernatant derived from 1x10^6^ cells of each *C. albicans* strain was separated by SDS-PAGE and transferred to a membrane and developed with a polyclonal rabbit antiserum that identifies Sap 1, Sap2 and Sap3, followed by a HPR swine anti-rabbit serum as a secondary antibody. The 42 kDa Sap2 band was detected in culture supernatant derived from one low, two medium and one high Gpm1/Pra1 expressing isolate. The figure shows a representative experiment out of three performed.


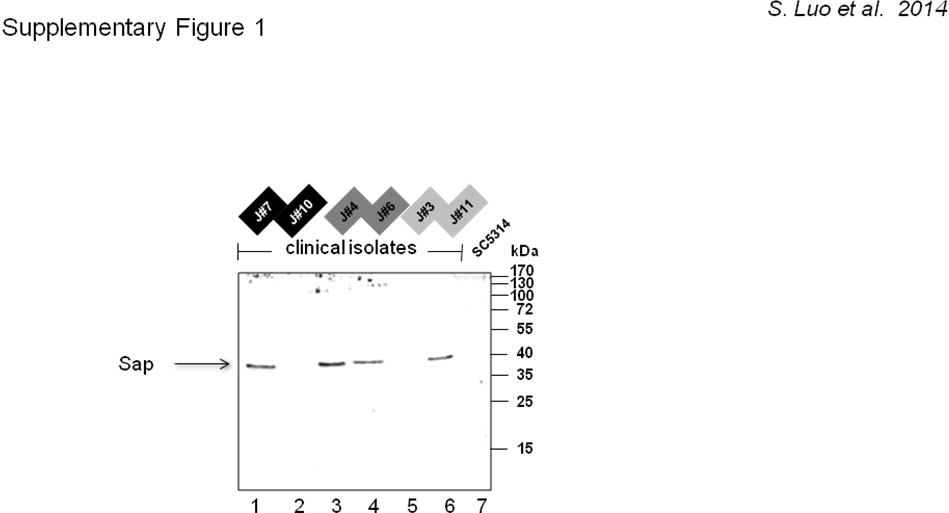

Supplement: S1 Fig — The selected C. albicans clinical strains and of the reference SC5314 strain were cultivated in YPD medium overnight at 30°C. Culture supernatant derived from 1x10^6^ cells of each C. albicans strain was separated by SDS-PAGE and transferred to a membrane and developed with a polyclonal rabbit anti Sap1/Sap2/Sap3 serum, followed by a HPR swine anti-rabbit serum as a secondary antibody. The 42 kDa Sap2 band was detected in culture supernatant derived from one low, two medium and one high Gpm1/Pra1 expressing isolate. The figure shows a representative experiment out of three performed. (DOC) [file pone.0113192.s001.doc]
